# Optimal Surgical Timing and Outcome Prediction in Hemorrhagic Moyamoya Disease: A Retrospective Cohort Study

**DOI:** 10.1002/cns.70917

**Published:** 2026-05-09

**Authors:** Qingbao Guo, Manli Xie, Zhengxing Zou, Qian‐Nan Wang, Cong Han, Xiangyang Bao, Lian Duan

**Affiliations:** ^1^ Department of Neurosurgery XI'AN NO. 9 HOSPITAL Xi'an Shaanxi China; ^2^ Department of Occupational Diseases Xi'an Central Hospital Xi'an Shaanxi China; ^3^ Department of Neurosurgery The Fifth Medical Centre, Chinese PLA General Hospital Beijing China; ^4^ Department of Neurosurgery The Eighth Medical Centre, Chinese PLA General Hospital Beijing China; ^5^ Department of Neurosurgery The First Medical Centre, Chinese PLA General Hospital Beijing China

**Keywords:** decision‐making, Encephaloduroarteriosynangiosis, hemorrhagic MMD, long‐term unfavorable outcomes, nomogram

## Abstract

**Objective:**

To develop and validate a prognostic nomogram incorporating optimal surgical timing thresholds for predicting long‐term outcomes in adult hemorrhagic moyamoya disease (MMD) patients undergoing encephaloduroarteriosynangiosis (EDAS).

**Methods:**

We conducted a retrospective cohort study of 256 consecutive adults with hemorrhagic MMD treated with EDAS (2013–2017) at a tertiary neurosurgical center. Using least absolute shrinkage and selection operator (LASSO) regression with 10‐fold cross‐validation, we identified key predictors for nomogram development. The model's performance was rigorously evaluated through discrimination (C‐index, AUC‐ROC), calibration, and decision curve analysis (DCA) in both training (70%) and validation (30%) cohorts.

**Results:**

Our hemorrhage‐specific nomogram incorporated five independent predictors: (1) age at initial hemorrhage, (2) surgical timing (optimal 3–6 month window post‐ictus), (3) preoperative functional status (modified Rankin Scale), (4) perioperative complications, and (5) recurrent hemorrhage. The model demonstrated excellent discrimination (C‐index: 0.889 training, 0.819 validation) and calibration. DCA confirmed superior clinical utility across 15%–80% probability thresholds compared to conventional approaches. Restricted cubic spline analysis validated the non‐linear relationship between surgical timing and outcomes.

**Conclusions:**

This study provides the first validated clinical decision tool for adult hemorrhagic MMD, establishing 3–6 months post‐hemorrhage as the optimal surgical window for EDAS. The nomogram's robust performance metrics and RCS‐validated temporal risk stratification offer neurosurgeons an evidence‐based approach to optimize revascularization timing and improve functional outcomes.

## Graphical abstract

This cohort study developed and validated the first prognostic nomogram for hemorrhagic MMD, incorporating five independent predictors including a defined optimal surgical window of 3 to 6 months. The model demonstrated good discrimination and aids in risk stratification for clinical monitoring. Key predictors include age at hemorrhage, preoperative functional status, and hemorrhage history.

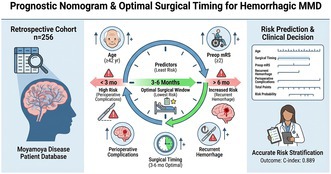

## 1 Introduction

Moyamoya disease (MMD) is a rare, progressive cerebrovascular disorder characterized by chronic stenosis or occlusion of the intracranial internal carotid arteries and their proximal branches, with compensatory formation of fragile collateral vessels [1]. These abnormal vascular networks predispose patients to both ischemic and hemorrhagic strokes, with hemorrhagic presentations accounting for 20%–40% of cases in Asian populations [2, 3]. Notably, hemorrhagic MMD carries a substantially higher risk of mortality and long‐term disability compared to its ischemic counterpart [4].

While surgical revascularization is widely accepted for ischemic MMD, the optimal management strategy for hemorrhagic MMD remains controversial [5, 6]. Indirect bypass techniques, such as encephaloduroarteriosynangiosis (EDAS), have demonstrated favorable angiographic and clinical outcomes in several studies [7, 8, 9, 10], yet the Japan Adult Moyamoya (JAM) trial reported superior rebleeding prevention with direct bypass over conservative management [11, 12]. This discrepancy underscores the lack of consensus on surgical selection and timing, highlighting the need for refined risk stratification tools to guide clinical decision‐making.

To address this gap, we developed the first nomogram integrating key clinical variables to predict long‐term adverse outcomes following EDAS in hemorrhagic MMD. Our model not only identifies critical prognostic factors but also provides preliminary insights into optimal surgical timing. By offering an evidence‐based predictive tool, this study aims to enhance personalized risk assessment and support neurosurgeons in tailoring revascularization strategies for high‐risk patients.

## 2 Methods

### 2.1 Ethics Approval and Data Availability

This retrospective study was conducted in accordance with the principles of the Declaration of Helsinki and approved by the local Institutional Review Board and Ethics Committee, with a waiver of informed consent due to the study's retrospective design. The reporting of this study follows the STROCSS (Strengthening the Reporting of Cohort Studies in Surgery) guidelines [13]. The datasets used and analyzed in this study are available from the corresponding author upon reasonable request.

### 2.2 Sample Size

Given a known sample proportion (*p*) of 20% [14], a margin of error (*δ*) of 0.06 for this survey, and a Type I error (*α*) set at 0.05 for hypothesis testing, the sample size is calculated using the formula: $n={\left(\frac{z_{1}-\alpha /2}{\delta }\right)}^{2}p\left(1-p\right)$, where z₁₋α/2 = 1.96. This yields a sample size of 171. Assuming a loss‐to‐follow‐up rate of 20%, the minimum required sample size is 214. In this study, a total of 256 patients were enrolled, which exceeds the calculated requirement and ensures statistical robustness.

### 2.3 Study Design and Patient Selection

We performed a single‐center retrospective cohort study of consecutive adult patients (≥ 18 years) with hemorrhagic MMD who underwent EDAS between January 2013 and December 2017 and completed ≥ 5 years of postoperative follow‐up. The diagnosis of MMD was confirmed by digital subtraction angiography (DSA) according to established guidelines [15]. Inclusion required: [1] Radiologically confirmed hemorrhagic presentation preceding surgery; [2] Treatment with unilateral or bilateral EDAS (only initial surgical hemispheres analyzed); [3] Complete clinical and imaging follow‐up data; and [4] having undergone surgical interventions (e.g., ventricular drainage or hematoma evacuation) for the acute qualifying hemorrhagic event. Exclusion criteria included: [1] Secondary moyamoya syndrome associated with systemic diseases (e.g., CNS tumors, severe brain trauma); [2] Previous craniotomy or revascularization procedures.

### 2.4 Data Collection and Outcome Measures

We systematically analyzed demographic, clinical, and radiographic variables previously associated with MMD outcomes, including: Demographic factors: sex, age (stratified at 42 years based on ROC analysis; Figure S1); Clinical characteristics: onset with symptoms, time from hemorrhage to surgery, bleeding episodes, comorbidities (hypertension [16], diabetes [7], hyperlipidemia, hyperhomocysteinemia [16]); Radiographic features: Suzuki stage, vascular anomalies (aneurysms, dilated anterior choroidal (AChA) or posterior communicating (PCoA)); Functional outcomes: modified Rankin Scale (mRS) scores at last follow‐up (unfavorable outcome defined as mRS ≥ 2) [17]. For the purpose of the primary long‐term functional outcome analysis, only patients with a minimum follow‐up duration of 5 years were included. An unfavorable outcome was defined as a modified Rankin Scale (mRS) score of ≥ 2 at the last available clinical follow‐up at or after the 5‐year minimum follow‐up milestone. Perioperative complications were defined as any surgical or medical adverse events occurring during the initial hospital stay for revascularization surgery or within 30 days postoperatively.

Two blinded neurosurgeons independently reviewed all clinical records and DSA studies, with discrepancies resolved through consensus. Symptom classification followed standardized criteria: Cerebral infarction required both clinical evidence and MRI confirmation; Hemorrhagic presentation was based on radiographic evidence regardless of onset with symptoms; Comorbidities were defined per established guidelines. This rigorous methodology ensured consistent data collection and minimized potential confounding factors in our analysis of EDAS outcomes for hemorrhagic MMD.

### 2.5 Surgical Implementation

All EDAS procedures were performed by the same neurosurgical team using a standardized technique [18]. The key surgical steps involved careful harvesting of the superficial temporal artery with its surrounding galea cuff while preserving both distal and proximal arterial segments, followed by precise grafting of the vascular pedicle into a linear dural opening created during osteotomy. This meticulous approach ensured optimal vascular preservation and subsequent collateral formation while maintaining surgical consistency across all cases.

### 2.6 Radiological Assessment

All hemispheres were angiographically classified using Suzuki's six‐stage grading [1] system based on routine DSA. Vascular anomalies were specifically assessed for the presence of dilated AChA or PCoA arteries, defined as either vessel demonstrating distal branching abnormalities or serving as collateral pathways [7]. These morphological features were considered significant markers of hemorrhagic risk, as established in previous studies [19]. The standardized evaluation protocol ensured consistent radiographic assessment across all cases while maintaining focus on clinically relevant vascular abnormalities associated with bleeding propensity.

### 2.7 Timing of Surgery

The determination of optimal surgical timing for hemorrhagic MMD integrated multiple patient‐specific factors, including hemorrhage severity, neurological status, comorbidities, and age. Among these, the interval between bleeding episodes was a critical determinant: expedited surgery was pursued after stabilization for single bleeds or those with a > 2‐month interval, whereas a deliberate delay until full stabilization was implemented for rapid recurrences (< 2‐month interval). Although early revascularization may theoretically reduce rebleeding risks [20], clinical realities such as acute stroke or systemic infections often necessitate surgical delay [21], while Chinese consensus guidelines recommend EDAS within 1–3 months following hemorrhage stabilization [22]. Existing evidence suggests early intervention (≤ 6 months) carries increased risks of perioperative seizures and wound complications compared to delayed procedures (> 6 months) [23]. Our selection of 6 months as a critical timepoint reflects both the established neuroplasticity window for functional recovery and the practical requirements for neurological stabilization through systematic rehabilitation [24, 25]. Based on these considerations and the natural history of hemorrhage recovery, we preliminarily explored the impact of surgical timing (stratified as < 3 months, 3–6 months, and > 6 months post‐ictus) on clinical outcomes in MMD.

### 2.8 Clinical Follow‐Up

Long‐term outcomes were systematically assessed through standardized clinic visits supplemented by telephone or mail interviews when necessary. An experienced physician conducted all evaluations using a predefined protocol to ensure consistency. The follow‐up protocol included quarterly assessments during the first postoperative year, transitioning to biannual evaluations in the second year, and annual reviews thereafter, providing comprehensive longitudinal data while maintaining patient convenience and compliance.

### 2.9 RCS Analysis of Age and Operative Timing Effects

To explore the nonlinear association between the duration from the initial bleeding to the operation, age, and adverse outcomes using restricted cubic splines (RCS) in the context of a clinical study. In this study, we collected data on adverse outcomes, the continuous predictor variable duration from the initial bleeding to the operation, and age. Possible nonlinear relationships between the change in duration from the initial bleeding to the operation, age, and adverse outcomes were examined by a logistic regression model with RCS.

Given our sample size and to balance flexibility with parsimony in the multivariable model, 4 knots were placed at the 5th, 35th, 65th, and 95th centiles to flexibly model the association, which is consistent with the approach used in prior studies [26].

### 2.10 Statistical Analysis

We implemented a randomized cohort allocation design to divide the study population (*n* = 256) into training (70%) and validation (30%) sets using stratified sampling based on outcome variables. The partitioning was performed in R (version 4.2.3) using the createDataPartition function with caret package (version 6.0–94) with a fixed random seed (1234) for reproducibility, ultimately yielding 179 (69.92%) training cases and 77 (30.08%) validation cases. The normality of all continuous data was formally assessed using the Shapiro–Wilk test. Based on the results, normally distributed data were analyzed with parametric tests (Student's *t*‐test), while non‐normally distributed data were analyzed with their non‐parametric equivalents (Mann–Whitney U test), with group comparisons performed using χ [2]/Fisher's exact tests for categorical variables. Variable selection was performed using Least Absolute Shrinkage and Selection Operator (LASSO) regression, implemented with the glmnet package (version 4.1–8) in *R*. The optimal penalty parameter (lambda, λ) was determined via 10‐fold cross‐validation minimizing the mean squared error. The selected features were then incorporated into a nomogram. The model's performance was assessed by evaluating its discrimination (via ROC analysis), calibration (with calibration plots), and clinical utility (using decision curve analysis). All analyses were conducted using MedCalc (version 20.218) and R, with a two‐tailed significance level of α = 0.05.

## 3 Results

### 3.1 Population Characteristics

**FIGURE 1:**
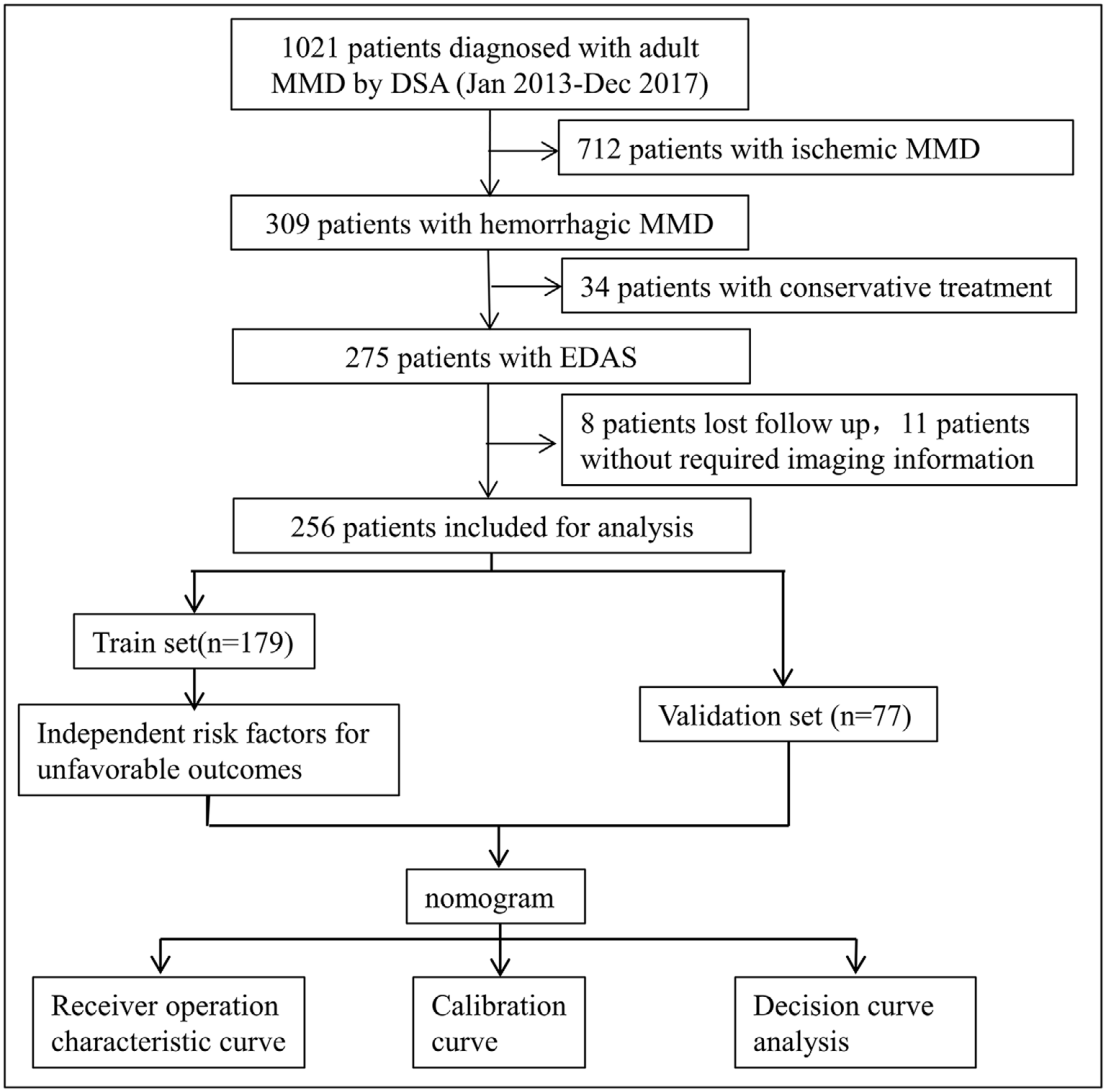
Flowchart of patient inclusion. DSA, digital subtraction angiography; MMD, moyamoya disease; EDAS, encephaloduroarteriosynangiosis.

Between 2013 and 2017, 1021 adult patients received DSA‐confirmed MMD diagnoses, including 309 (30.3%) hemorrhagic cases. After excluding 34 conservatively managed patients and 19 cases (11 with incomplete imaging, 8 lost to follow‐up), our final cohort comprised 256 EDAS‐treated patients (Figure 1). Perioperative complications occurred in 8 cases (3.1%), including cerebral infarction (*n* = 2), cerebellar hemorrhage (*n* = 2), and other hemorrhagic events (*n* = 4). During a median follow‐up of 7.51 years (range, 5.00–10.15), 13 patients (5.1%) experienced rebleeding, one of which was fatal.

The cohort was randomly allocated into training (*n* = 179) and validation (*n* = 77) sets with balanced baseline characteristics (Table 1). Both groups showed comparable distributions of sex (57.8% female), clinical presentation, surgical timing (38.7% operated ≤ 3 months post‐hemorrhage), treatment approaches, bleeding frequency, Suzuki stages, preoperative functional status (predominantly mRS 0), and comorbidities including hypertension, hyperlipidemia, diabetes, and hyperhomocysteinemia. Vascular characteristics (5.9% aneurysm prevalence, dilated AChA/PCoA) also demonstrated intergroup equivalence (all *p* > 0.05).

**TABLE 1:** Patient demographics and baseline characteristics in the train and validation set.

Characteristic	Cohort			*p* ^2^
	Overall, *N* = 256^1^	Train set, *N* = 179^1^	Validation set, *N* = 77^1^	
Gender				0.887
Female	148 (57.8%)	104 (58.1%)	44 (57.1%)	
Male	108 (42.2%)	75 (41.9%)	33 (42.9%)	
Age (years)				0.683
﹤ 42	128 (50.0%)	91 (50.8%)	37 (48.1%)	
≥ 42	128 (50.0%)	88 (49.2%)	40 (51.9%)	
Onset with symptoms				0.482
Cerebral infarction	8 (3.1%)	7 (3.9%)	1 (1.3%)	
Intraparenchymal hemorrhage	78 (30.5%)	56 (31.3%)	22 (28.6%)	
Other	14 (5.5%)	10 (5.6%)	4 (5.2%)	
Subarachnoid hemorrhage	24 (9.4%)	13 (7.3%)	11 (14.3%)	
TIA	19 (7.4%)	12 (6.7%)	7 (9.1%)	
Ventricular hemorrhage	113 (44.1%)	81 (45.3%)	32 (41.6%)	
Onset‐operation (months)				0.937
Mean ± SD	7.45 ± 2.35	7.52 ± 2.55	7.42 ± 3.01	
< 3	99 (38.7%)	69 (38.5%)	30 (39.0%)	0.824
3–6	66 (25.8%)	48 (26.8%)	18 (23.4%)	
≥ 6	91 (35.5%)	62 (34.6%)	29 (37.7%)	
Treatment after cerebral hemorrhage				0.430
Conservative treatment	167 (65.2%)	113 (63.1%)	54 (70.1%)	
External drainage of hematoma via ventricular puncture	138 (53.9%)	95 (53.1%)	43 (55.8%)	
Hematoma Evacuation through Craniotomy	29 (11.3%)	18 (10.1%)	11 (14.3%)	
Number of episodes of bleeding				0.265
1	242 (94.5%)	171 (95.5%)	71 (92.2%)	
2	12 (4.7%)	6 (3.4%)	6 (7.8%)	
3	2 (0.8%)	2 (1.1%)	0 (0.0%)	
Suzuki stage				0.442
﹤IV	139 (54.3%)	100 (55.9%)	39 (50.6%)	
≥ IV	117 (45.7%)	79 (44.1%)	38 (49.4%)	
Fellow‐up (years)				0.983
Mean ± SD	7.46 ± 1.36	7.46 ± 1.37	7.46 ± 1.35	
Preoperative				0.343
mRS				
﹤2	172 (67.2%)	117 (65.4%)	55 (71.4%)	
≥ 2	84 (32.8%)	62 (34.6%)	22 (28.6%)	
Hypertension				0.211
No	193 (75.4%)	131 (73.2%)	62 (80.5%)	
Yes	63 (24.6%)	48 (26.8%)	15 (19.5%)	
Hyperlipidemia				0.697
No	229 (89.5%)	161 (89.9%)	68 (88.3%)	
Yes	27 (10.5%)	18 (10.1%)	9 (11.7%)	
Diabetes				> 0.999
No	247 (96.5%)	173 (96.6%)	74 (96.1%)	
Yes	9 (3.5%)	6 (3.4%)	3 (3.9%)	
Hyperhomocysteine				0.627
no	239 (93.4%)	168 (93.9%)	71 (92.2%)	
yes	17 (6.6%)	11 (6.1%)	6 (7.8%)	
Recurrent bleeding during follow‐up				0.355
Yes	13 (5.1%)	11 (6.1%)	2 (2.6%)	
No	243 (94.9%)	168 (93.9%)	75 (97.4%)	
Perioperative complications				0.585
Cerebral Infarction	2 (0.8%)	2 (1.1%)	0 (0.0%)	
Cerebral parenchymal hemorrhage	2 (0.8%)	1 (0.6%)	1 (1.3%)	
Epidural hematoma	1 (0.4%)	0 (0.0%)	1 (1.3%)	
Intraventricular hemorrhage	1 (0.4%)	1 (0.6%)	0 (0.0%)	
Subarachnoid hemorrhage	2 (0.8%)	2 (1.1%)	0 (0.0%)	
No	248 (96.9%)	173 (96.6%)	75 (97.4%)	
Presence of aneurysm				0.442
No	248 (96.9%)	172 (96.1%)	76 (98.7%)	
Yes	8 (3.1%)	7 (3.9%)	1 (1.3%)	
AChA dilation				0.710
No	237 (92.6%)	165 (92.2%)	72 (93.5%)	
Yes	19 (7.4%)	14 (7.8%)	5 (6.5%)	
PCoA dilation				0.621
No	215 (84.0%)	149 (83.2%)	66 (85.7%)	
Yes	41 (16.0%)	30 (16.8%)	11 (14.3%)	

^1^ *n* (%).

^2^ Pearson's Chi‐squared test; Fisher's exact test; Welch Two Sample *t*‐test. AChA, anterior choroidal artery; mRS, modified Rankin scale; PCoA, posterior communicating artery.

### 3.2 Operation Time

**FIGURE 2:**
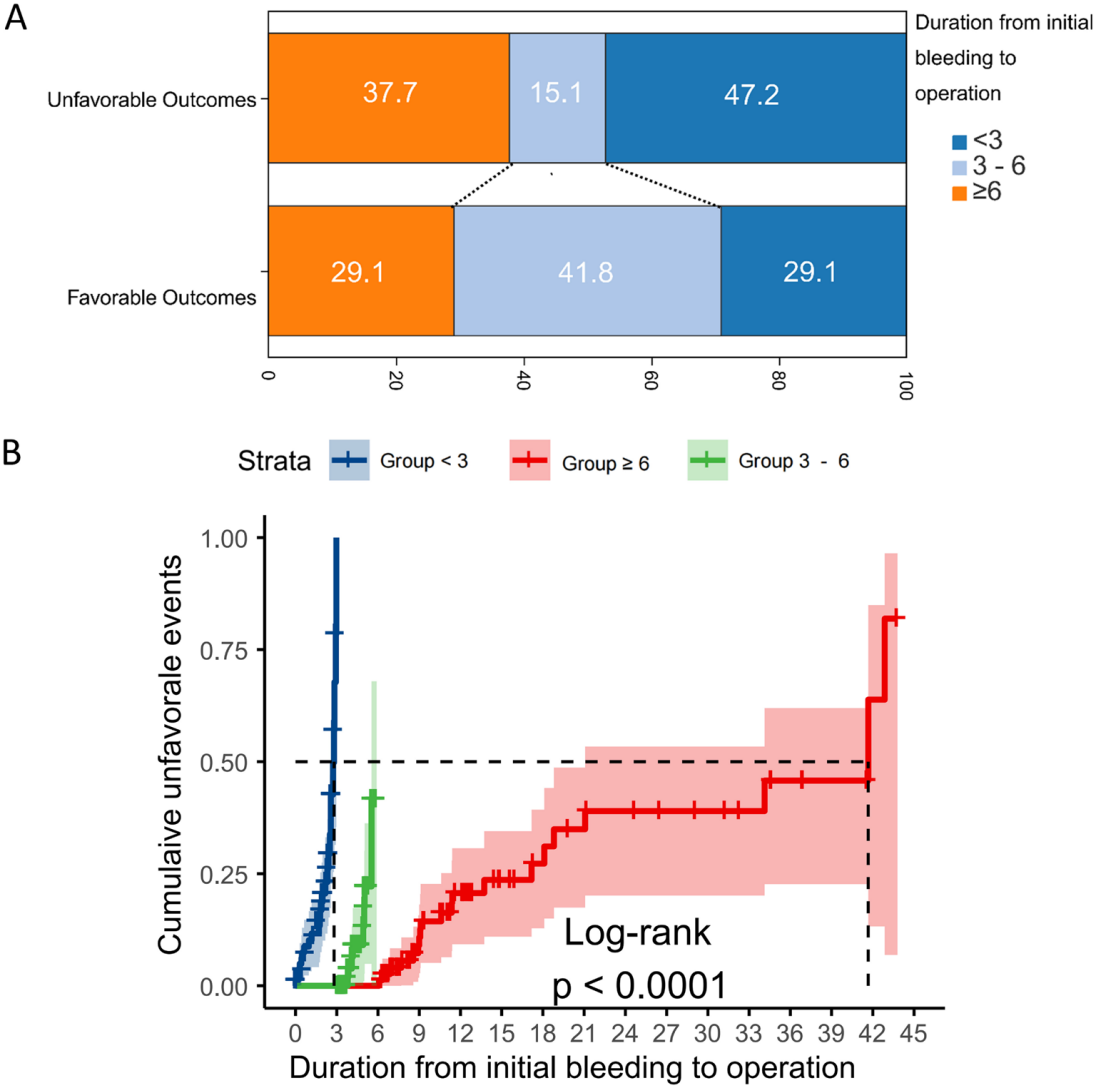
Operation time. (A) Outcomes based on the duration from the initial bleeding to the operation (< 3 months, 3–6 months, and > 6 months). (B) Kaplan–Meier curve illustrating the poor prognosis probabilities among patients undergoing surgeries at < 3 months, 3–6 months, and > 6 months.

Patients undergoing surgery during the 3–6 month post‐hemorrhage window demonstrated significantly better outcomes, with a 41.8% favorable outcome rate compared to 47.2% in the ≤ 3 month group and 37.7% in the ≥ 6 month group (*p* < 0.05, Figure 2A). This optimal timing window was further supported by Kaplan–Meier analysis, which revealed significantly improved long‐term outcomes for the 3–6 month cohort relative to both earlier and delayed intervention groups (*p* < 0.05, Figure 2B).

### 3.3 Nomogram Variable Screening

**FIGURE 3:**
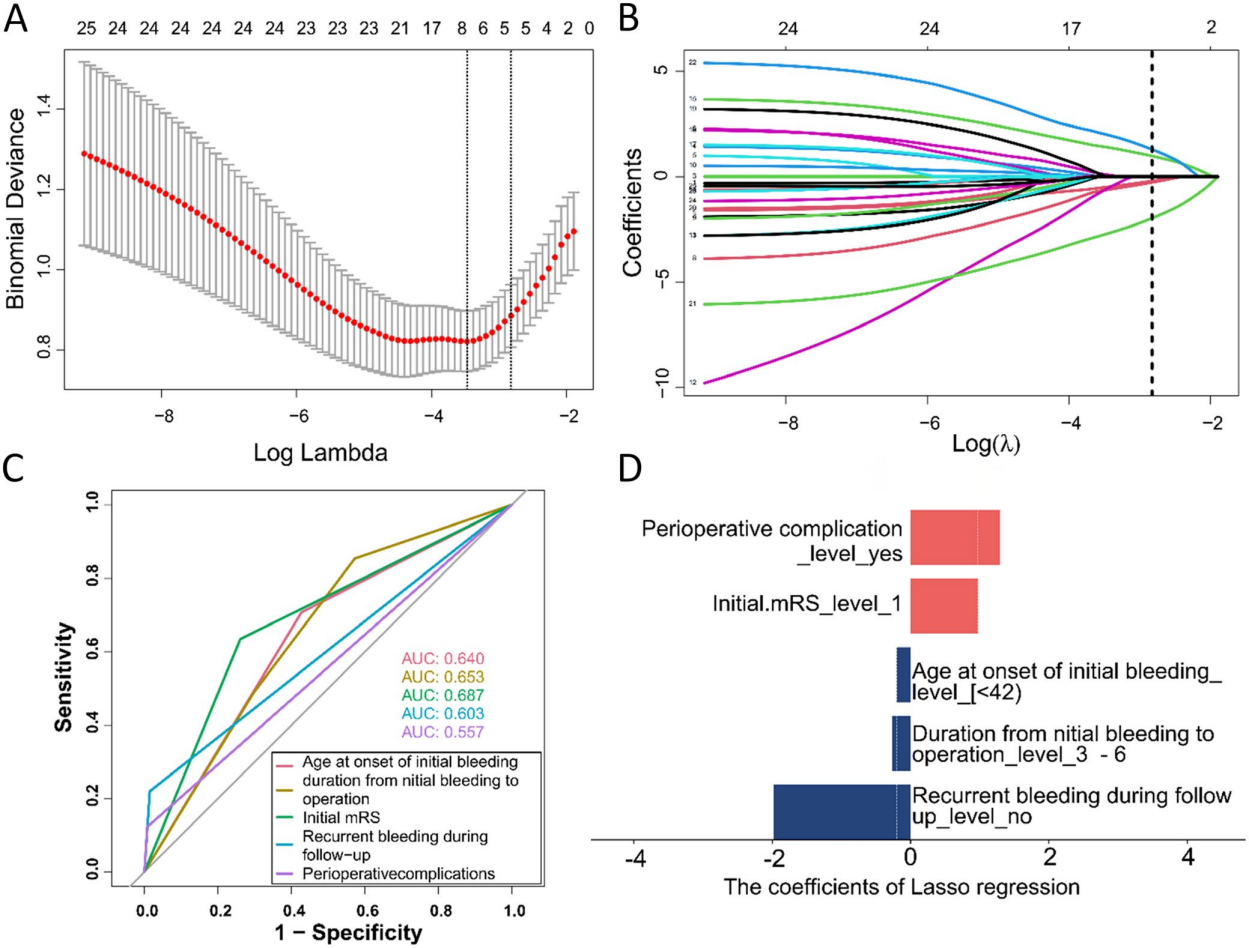
Nomogram variable screening. (A) LASSO coefficient paths for the 19 initial variables. The left vertical dashed line indicates the log λ value at the minimum cross‐validation error (λ. min), while the right vertical dashed line indicates the log λ value at the most regularized model within one standard error of the minimum (λ.1se), where 5 non‐zero coefficients were retained. (B) Cross‐validation deviance plot for LASSO regression. The vertical dashed line represents the optimal λ selected by the one standard error rule (λ.1se), balancing model fit and parsimony. (C) Receiver operating characteristic curve analysis of the five candidate risk indicators. (D) The LASSO coefficients for the selected variables. AUC, area under the time‐dependent receiver operating characteristic curve; LASSO, least absolute shrinkage and selection operator. AUC, area under the time‐dependent receiver operating characteristic curve; LASSO, least absolute shrinkage and selection operator.

The initial comprehensive model incorporated 18 clinical variables encompassing demographic characteristics (sex, age at initial hemorrhage), disease severity markers (bleeding episodes, Suzuki stage), functional status (preoperative mRS), comorbidities (hypertension, diabetes), vascular anomalies (aneurysms, dilated AChA/PCoA), and treatment‐related factors (surgical timing, perioperative complications). The optimal tuning parameter (*λ*) was chosen using the one standard error rule, yielding a parsimonious model that retained five variables: age at onset of initial bleeding, duration from initial bleeding to operation of 3–6 months, preoperative mRS, recurrent bleeding during follow‐up, and perioperative complications (Figure 3A). Figure 3B presents the cross‐validation deviance plot. The discriminative ability of these individual predictors, as assessed by area under the receiver operating characteristic curve, ranged from 0.557 to 0.687 (Figure 3C), and the magnitude of their LASSO regression coefficients reflected the relative contribution of each variable to the prediction model (Figure 3D).

### 3.4 Multivariate Logistic Regression for the Training Cohort

Multivariate logistic regression identified five independent predictors of long‐term unfavorable outcomes (Table 2): advanced age at initial hemorrhage (OR 0.38, 95% CI 0.15–0.97; *p* = 0.043), surgical intervention during the 3–6 month post‐bleeding window (OR 0.17, 95% CI 0.05–0.60; *p* = 0.006), poorer preoperative functional status (mRS OR 11.49, 95% CI 3.89–33.94; *p* < 0.001), recurrent hemorrhage during follow‐up (OR 71.34, 95% CI 11.12–457.79; *p* < 0.001), and perioperative complications (OR 36.44, 95% CI 2.95–449.60; *p* = 0.006). These results demonstrate particularly strong associations for recurrent bleeding and surgical complications, while confirming the importance of the previously identified optimal surgical timing window.

**TABLE 2:** Multivariate logistic regression analyses assessing the long‐term unfavorable outcomes after EDAS for training cohort.

Characteristic	*N*	Event *N*	OR	95% CI	*p*
Age at onset of initial bleeding (years)					
< 42	91	12	—	—	
≥ 42	88	29	2.66	1.03, 6.86	0.043
Duration from initial bleeding to operation (months)					
< 3	61	20	—	—	
3–6	65	6	0.17	0.05, 0.60	0.006
≥ 6	53	15	0.38	0.13, 1.11	0.077
Preoperative mRS					
< 2	117	15	—	—	
≥ 2	62	26	11.49	3.89, 33.94	< 0.001
Recurrent bleeding during follow‐up					
No	168	32	—	—	
Yes	11	9	71.34	11.12, 457.79	< 0.001
Perioperative complications					
No	173	36	—	—	
Yes	6	5	36.44	2.95, 449.60	0.005

Abbreviations: CI, Confidence Interval; OR, Odds Ratio.

### 3.5 Nomogram Construction and Validation

**FIGURE 4:**
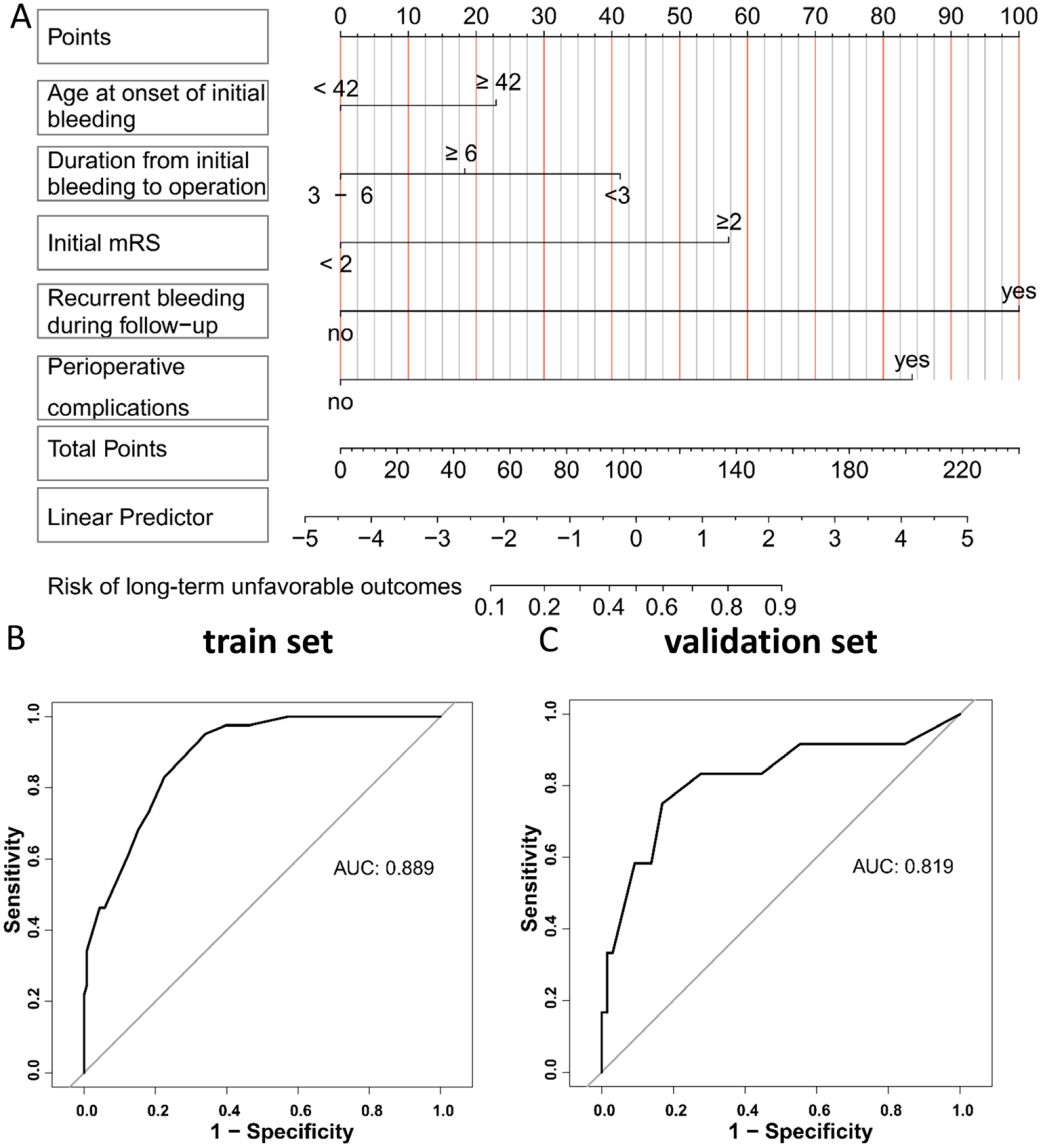
Nomogram construction and validation. (A) A constructed nomogram predicting long‐term unfavorable outcomes following EDAS in adult patients with hemorrhagic MMD. (B, C) AUCs of the model in the training and validation set. AUC, area under the time‐dependent receiver operating characteristic curve; MMD, moyamoya disease; EDAS, encephaloduroarteriosynangiosis.

**FIGURE 5:**
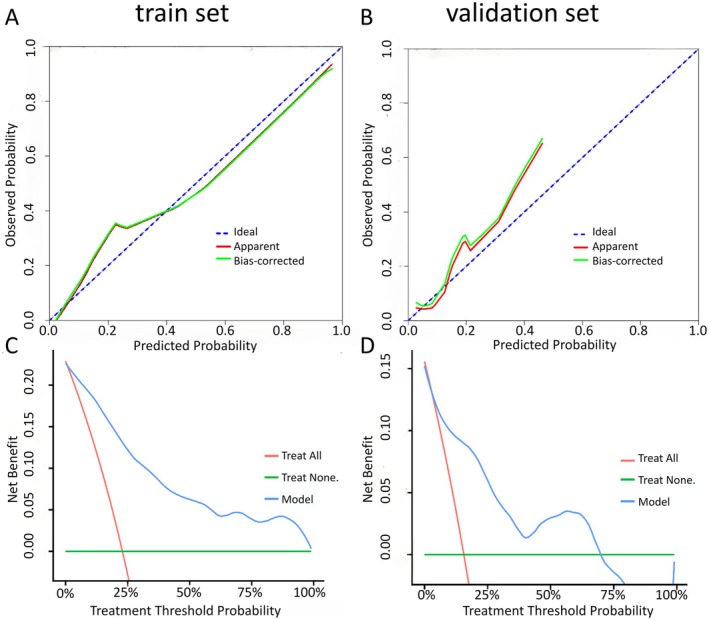
Decision curve analysis of the nomogram. Calibration curves of long‐term unfavorable outcomes prediction for adult patients with hemorrhagic MMD in the (A) training set and (B) validation set. Decision curve analysis of long‐term unfavorable outcomes prediction for adult patients with hemorrhagic MMD in the (C) training set and (D) validation set. MMD, moyamoya disease.

We developed a clinically applicable nomogram (Figure 4A) incorporating the five identified predictors, with total scores ranging 0–240 across the cohort. The model demonstrated excellent discrimination, with C‐indices of 0.889 (95% CI 0.840–0.938) in the training set and 0.819 (95% CI 0.660–0.979) in validation, supported by AUCs > 0.8 in both cohorts (Figure 4B,C). Calibration plots revealed strong agreement between predicted and observed outcomes in training and validation sets (Figure 5A,B), with curves closely approximating the ideal line. Decision curve analysis confirmed clinical utility across threshold probabilities of 10%–70% (Figure 5C,D), showing substantial net benefit for clinical decision‐making in both cohorts. These results collectively validate the nomogram as a reliable tool for predicting long‐term unfavorable outcomes in hemorrhagic MMD patients following EDAS.

### 3.6 RCS Analysis of Age and Operative Timing Effects

**FIGURE 6:**
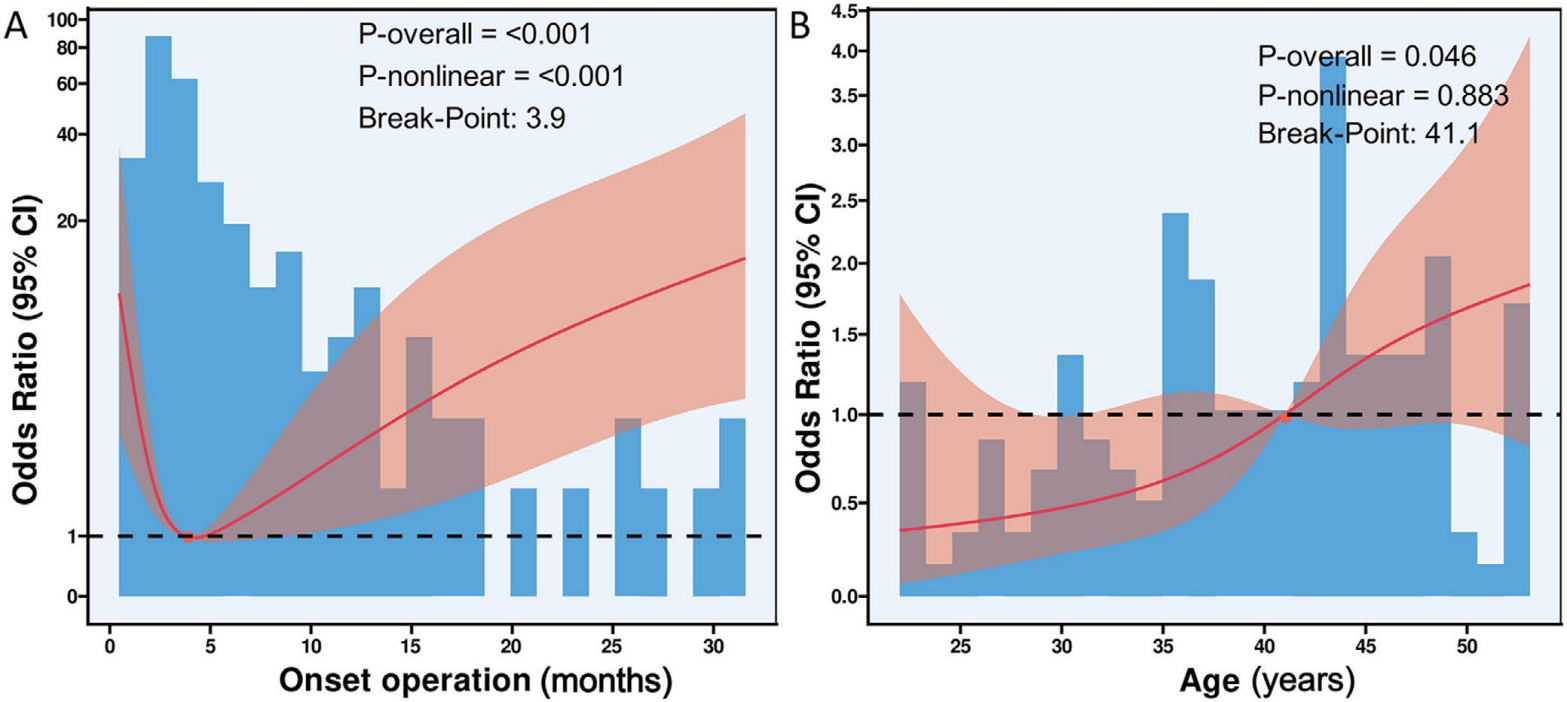
Association between the duration from the initial bleeding to the operation, age, and adverse outcomes using the restricted cubic spline (RCS) function. (A) Association between duration from the initial bleeding to the operation and adverse outcomes with the RCS function. (B) Association between age and adverse outcomes with the RCS function. Model with 4 knots located at the 5th, 35th, 65th, and 95th percentiles.

RCS Analysis demonstrated a significant nonlinear relationship between surgical timing and poor outcomes (P‐nonlinear < 0.001), with risk initially decreasing sharply from hemorrhage onset, reaching its lowest point at 3.5–5 months, then progressively increasing with longer delays (P‐overall < 0.001; Figure 6A). In contrast, age showed a linear positive association with poor outcomes (P‐overall = 0.046) without significant nonlinearity (P‐nonlinear = 0.883; Figure 6B). These differential patterns reveal important temporal dynamics in post‐hemorrhagic MMD outcomes, with surgical timing demonstrating a clear optimal window while age exhibits simpler cumulative risk effects.

## 4 Discussion

Our study establishes that 20.7% of hemorrhagic MMD patients experience unfavorable long‐term outcomes (mRS ≥ 2) following EDAS. Multivariable analysis identified five clinically modifiable predictors: (1) advanced age (≥ 42 years at initial hemorrhage), (2) poorer baseline function (admission mRS ≥ 2), (3) suboptimal surgical timing (< 3 or ≥ 6 months post‐ictus), (4) perioperative complications, and (5) hematoma recurrence. These findings were incorporated into a practical nomogram that enables: [1] preoperative risk quantification, [2] evidence‐based surgical timing decisions, and [3] postoperative monitoring prioritization for high‐risk patients. The model's discrimination (C‐index 0.889) and calibration performance support its clinical implementation for personalized management of hemorrhagic MMD.

Although revascularization surgery has become well‐established for ischemic MMD based on robust clinical evidence [5, 6], the optimal therapeutic strategy for hemorrhagic presentations continues to generate considerable debate [4, 27]. The JAM trial provided the first randomized controlled evidence supporting direct bypass over conservative management for reducing rebleeding risk and improving clinical outcomes in hemorrhagic MMD [28]. Despite this advance, critical gaps persist in our understanding of long‐term comparative effectiveness between direct and indirect revascularization approaches, particularly regarding their durability and mechanism‐specific outcomes. This knowledge deficit underscores the pressing need for studies that clarify the distinct therapeutic roles of various surgical strategies in hemorrhagic MMD management.

Revascularization surgery remains the cornerstone of hemorrhagic MMD treatment, addressing both cerebral hypoperfusion and rebleeding risk through distinct temporal mechanisms. While traditional paradigms emphasize the gradual development of neocollateral vessels over 3–4 months post‐EDAS [29], contemporary neuroimaging revelations have fundamentally reshaped this timeline. Advanced perfusion techniques including arterial spin labeling (ASL‐MRI) and dynamic susceptibility contrast‐enhanced perfusion‐weighted imaging (DSC‐PWI) now demonstrate measurable hemodynamic improvements emerging as early as 2–4 weeks postoperatively [30], with progressive enhancement through the critical 3‐month window. This early perfusion augmentation precedes macroscopic collateral formation and likely mediates clinical benefit through two synergistic mechanisms: immediate restoration of cerebral blood flow to ischemic territories and reduction of pathological shear stress in fragile moyamoya vessels [31]. The resultant hemodynamic stabilization appears particularly crucial for hemorrhage prevention, as normalized wall stress may prevent rupture of tenuous collateral networks even before complete angiographic revascularization occurs.

EDAS has emerged as a particularly advantageous revascularization approach, offering superior procedural safety and technical feasibility for high‐risk patients with significant comorbidities or compromised donor vasculature [32, 33]. At our institution, EDAS demonstrates remarkable clinical efficacy, achieving durable symptom resolution in 95% of treated patients across both adult and pediatric populations [34]. The procedure's effectiveness stems from its unique dual‐mechanism action: it induces robust neoangiogenesis through collateral networks derived from the superficial temporal and middle meningeal arteries while also establishing direct anastomoses between these extracranial vessels and cortical branches [35, 36]. Importantly, longitudinal outcomes data confirm EDAS's capacity to substantially mitigate rebleeding risk—a crucial therapeutic endpoint given the well‐documented association between advancing age and hemorrhage recurrence from dilated AChA and PCoA arteries [7].

The long‐term outcomes following EDAS for hemorrhagic MMD are governed by multiple factors, with recurrent hemorrhage standing out as the most critical prognostic variable across multiple studies [37, 38]. Our data reinforce this established clinical paradigm, demonstrating that rebleeding events trigger accelerated neurological decline, a finding consistent with Kang et al. report of 40% rebleeding rates strongly correlating with poor outcomes [37]. The primacy of rebleeding as an outcome determinant has been further validated in longitudinal analyses, including a study of 95 hemorrhagic MMD patients that identified recurrent hemorrhage as the dominant predictor of prognosis regardless of treatment approach [7]. These consistent observations underscore the progressive nature of hemorrhagic MMD and emphasize the clinical imperative for implementing rigorous, standardized surveillance protocols to identify and mitigate rebleeding risks in this vulnerable patient population.

Although not formally established as an independent prognostic factor, mounting evidence positions age at initial hemorrhage as a critical modulator of rebleeding risk following EDAS. Huang et al. [39] first documented this association, reporting increased rebleeding incidence with advancing age (mean 36.8 ± 10.1 years), a finding subsequently validated by Morioka et al. [40] who identified age > 36 years as predictive of reduced rebleeding‐free survival. Our data corroborate these observations while precisely defining the high‐risk threshold at ≥ 42 years, coinciding with the fifth to sixth decades of peak vascular vulnerability [7, 36]. The pathophysiological basis likely involves age‐related microvascular degeneration characterized by progressive luminal stenosis and mural fragility in perforating vessels, which collectively elevate hemodynamic stress within collateral networks beyond rupture thresholds.

Current guidelines strongly advocate revascularization surgery for hemorrhagic MMD to optimize outcomes [41], yet optimal intervention timing remains controversial. While early surgery (≤ 3 months) shows promise for reducing rebleeding and improving recovery [42, 43], conflicting data associate both acute‐phase and delayed (> 6 months) interventions with increased complications like seizures and wound issues [23, 44]. This therapeutic paradox highlights the intricate risk–benefit calculus required when determining surgical timing for hemorrhagic MMD patients.

Our analysis identifies 3–6 months post‐hemorrhage as the optimal surgical window, challenging conventional early intervention paradigms [15]. Acute‐phase surgeries (< 3 months) risk hemodynamic instability due to incomplete vascular stabilization, while delayed procedures (≥ 6 months) allow progressive collateral network deterioration. This defined interval enables essential inflammatory resolution while preventing secondary hemorrhage through timely revascularization of vulnerable vessels.

Preoperative neurological status independently predicts revascularization outcomes in MMD [45], with our data (20.7% unfavorable outcomes in 53/256 cases) showing a strong correlation between mRS ≥ 3 and poor prognosis. This aligns with Kawaguchi et al. [46] demonstration of dose‐dependent mRS‐morbidity relationships and Ha et al. [45] findings of better outcomes with mRS ≤ 2. Such consistency underscores the value of baseline mRS assessment for identifying patients with probable irreversible microvascular damage.

Perioperative complications, particularly hemorrhage (2.34%) and infarction (0.78%), emerge as independent predictors of long‐term outcomes in hemorrhagic MMD, extending beyond their established short‐term effects [47]. While our hemorrhage rates align with historical MMD cohorts (0.13%–3.6%) [48, 49], the underlying mechanisms remain incompletely understood. Ischemia–reperfusion injury may play a key role, with revascularization triggering endothelial activation and matrix metalloproteinase release [50] that disrupt blood–brain barrier integrity through both increased vascular permeability and structural junction degradation, explaining the delayed hemorrhage phenomenon.

RCS analysis revealed a significant nonlinear relationship (P‐nonlinear < 0.001) between surgical timing and outcomes, with risk nadiring at 3.5–5 months post‐hemorrhage. This validates our 3–6 month window as biologically optimal, showing steep risk reduction from ultra‐early (< 3 month) interventions and progressive deterioration with delays (> 6 months). The inflection points confirm these thresholds reflect true pathophysiological transitions rather than arbitrary divisions, identifying a distinct neuroprotective period where surgical benefits maximally outweigh risks.

This study makes three key advances in hemorrhagic MMD management: [1] development of the first validated nomogram for individualized outcome prediction, [2] identification of 3–6 months as a potentially optimal surgical window that helps reconcile previous conflicting reports by balancing hemorrhage prevention against procedural risks, and [3] demonstration of age‐specific outcomes supporting personalized timing approaches. While the single‐center retrospective design and potential unmeasured confounders warrant caution, our large cohort provides a robust foundation for future prospective studies to validate these findings and further refine surgical timing paradigms, potentially incorporating emerging biomarkers and genetic profiling to advance precision medicine in MMD care.

## 5 Limitations

This study presents four primary limitations. First, the single‐center design introduces inherent selection bias in EDAS patient enrollment, potentially restricting extrapolation of findings; external validation through multicenter nomogram verification remains critical. Second, protracted referral intervals associated with our tertiary center status resulted in advanced neurological deterioration at presentation, creating temporal heterogeneity in surgical timing that may influence outcome interpretation. Third, inconsistent preoperative cerebral perfusion assessment in early cohort patients constrains hemodynamic correlation analyses. Fourth, comparative efficacy evaluation of direct versus combined revascularization strategies for EDAS‐ineligible hemorrhagic cases remains underway. Additionally, unmeasured confounders—including intrinsic disease progression rates—may systematically affect outcome metrics. Residual prognostic influences from comorbid metabolic disorders (diabetes, hypertension), and uncharted MMD‐associated genetic polymorphisms were not fully addressed in this retrospective paradigm.

## 6 Conclusions

This study provides the first validated clinical decision tool for adult hemorrhagic MMD, establishing 3–6 months post‐hemorrhage as the optimal surgical window for EDAS. The nomogram's robust performance metrics and RCS‐validated temporal risk stratification offer neurosurgeons an evidence‐based approach to optimize revascularization timing and improve functional outcomes.

## Author Contributions

L.D., X.B., and C.H. designed the study. Q.G., M.L.X., and Z.Z. wrote the manuscript and conducted statistical analysis. Q.‐N.W. contributed to the manuscript discussion, figures, and tables. All authors contributed to the article and approved the submitted version.

## Funding

This work was supported by the National Natural Science Foundation of China (82171280 and 82201451).

## Disclosure

The authors have nothing to report.

## Ethics Statement

This study was approved by the Institutional Review Board and Ethics Committee of the Fifth Medical Center of the Chinese PLA General Hospital (approval number: ky‐2020‐9‐22). Due to the study's retrospective nature, the requirement for informed consent was waived.

## Conflicts of Interest

The authors declare no conflicts of interest.

## Supporting information


**Figure S1:** Upon calculation, we established the cutoff age for poor prognosis in hemorrhagic MMD as 42 years old. Please review the attachment.

## Data Availability

Original data were generated and stored at the Fifth Medical Center of the People's Liberation Army of China (PLA) General Hospital. Data supporting these results may be obtained from the corresponding authors if the requirements are reasonable.
