# Supplementary material for: Optimal Surgical Timing and Outcome Prediction in Hemorrhagic Moyamoya Disease: A Retrospective Cohort Study
Source: CNS Neurosci Ther. 2026 May 9;32(5):e70917. doi: 10.1002/cns.70917 (PMC13156802; doi:10.1002/cns.70917)

Optimal Surgical Timing and Outcome Prediction in Hemorrhagic Moyamoya Disease: A retrospective cohort study

Qingbao Guo^1#*^, Manli Xie^2#^, Zhengxing Zou^3#^, Qian-Nan Wang^4^, Cong Han^3^, Xiangyang Bao^5*^, Lian Duan^5*^

Figure S1. Upon calculation, we established the cutoff age for poor prognosis in hemorrhagic MMD as 42 years old.


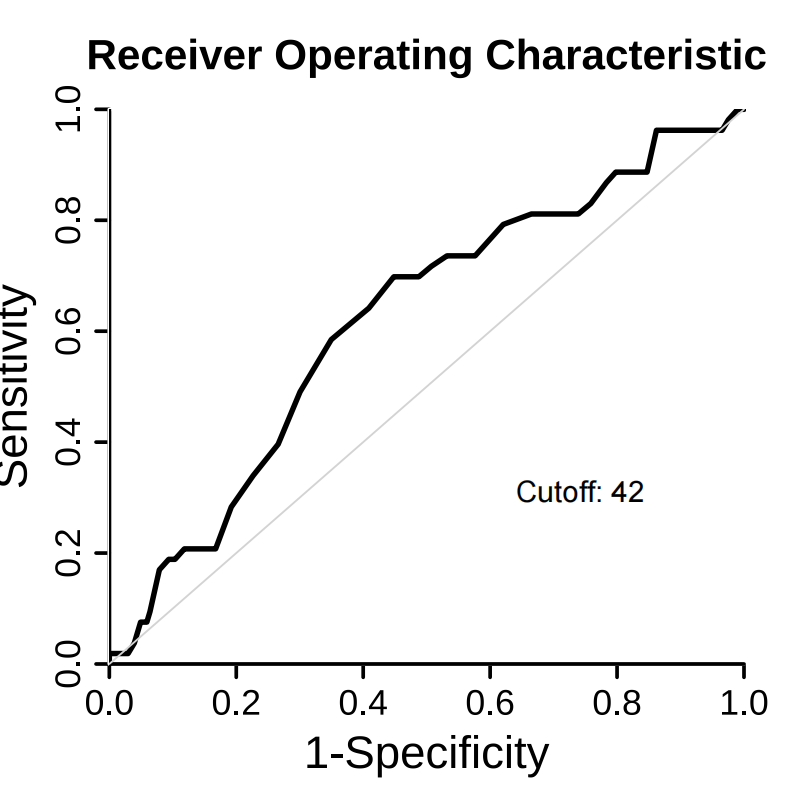

Supplement: Supplementary file 1 — Figure S1: Upon calculation, we established the cutoff age for poor prognosis in hemorrhagic MMD as 42 years old. Please review the attachment. [file CNS-32-e70917-s001.docx]
